# The Redox–Adhesion–Exosome (RAX) Hub in Cancer: Lipid Peroxidation-Driven EMT Plasticity and Ferroptosis Defense with HNE/MDA Signaling and Lipidomic Perspectives

**DOI:** 10.3390/antiox14121474

**Published:** 2025-12-08

**Authors:** Moon Nyeo Park, Jinwon Choi, Rosy Iara Maciel de Azambuja Ribeiro, Domenico V. Delfino, Seong-Gyu Ko, Bonglee Kim

**Affiliations:** 1 College of Korean Medicine, Kyung Hee University, 1-5 Hoegi-dong, Dongdaemun-gu, Seoul 02447, Republic of Korea; mnpark@khu.ac.kr (M.N.P.); wlsdnjsl6888@naver.com (J.C.); epiko@khu.ac.kr (S.-G.K.); 2 Korean Medicine-Based Drug Repositioning Cancer Research Center, College of Korean Medicine, Kyung Hee University, Hoegi-dong, Dongdaemun-gu, Seoul 02447, Republic of Korea; 3 Experimental Pathology Laboratory, Midwest Campus, Federal University of São João del-Rei, Divinópolis 35501-296, Brazil; rosy@ufsj.edu.br; 4 Department of Medicine and Surgery, Piazza Università 1, 06123 Perugia, Italy; domenico.delfino@unipg.it

**Keywords:** RAX hub, EMT plasticity, exosomal PD-L1/miRNAs, CDH2/N-cadherin, lipid peroxidation, 4-hydroxynonenal (HNE), malondialdehyde (MDA)

## Abstract

Cancer cell plasticity drives metastasis and therapy resistance through dynamic transitions between epithelial, mesenchymal, and neural crest stem-like (NCSC) states; however, a unifying mechanism that stabilizes these transitions remains undefined. To address this gap, we introduce a N-cadherin (CDH2)-centered redox–adhesion–exosome (RAX) hub that links oxidative signaling, adhesion dynamics, and exosome-mediated immune communication into a closed-loop framework. Within this network, reactive oxygen species (ROS) pulses license epithelial–mesenchymal transition (EMT), AXL–FAK/Src signaling consolidates mesenchymal adhesion, and selective exosomal cargoes—including miR-21, miR-200, miR-210, and PD-L1—propagate plasticity and immune evasion. Lipid peroxidation acts as a central checkpoint connecting ROS metabolism to PUFA membrane remodeling and ferroptosis vulnerability, buffered by NRF2–GPX4 and FSP1/DHODH axes, thereby converting transient oxidative pulses into persistent malignant states. Mechanistically, the RAX hub synthesizes findings from EMT/CSC biology, ferroptosis defenses, and exosome research into a self-reinforcing system that sustains tumor heterogeneity and stress resilience. Evidence from single-cell and spatial transcriptomics, intravital ROS imaging, and exosome cargo-selector studies supports the feasibility of this model. We further outline validation strategies employing HyPer–EMT–CDH2 tri-reporters, CRISPR perturbation of YBX1/ALIX cargo selectors, and spatial multi-omics in EMT-high tumors. Clinically, tumors enriched in EMT/NCSC programs—such as melanoma, neuroblastoma, small-cell lung cancer, pancreatic ductal adenocarcinoma, and triple-negative breast cancer (TNBC)—represent RAX-dependent contexts. These insights highlight biomarker-guided opportunities to target adhesion switches, ferroptosis defenses, and exosome biogenesis through lipid peroxidation-centered strategies using liquid-biopsy panels (exosomal CDH2, miR-200, miR-210) combined with organoid and xenograft models. By linking lipid peroxidation to ferroptosis defense and oxidative stress adaptation, the RAX hub aligns with the thematic focus of lipid metabolism and redox control in cancer progression. Collectively, the RAX framework may provide a conceptual basis for precision oncology by reframing metastasis and therapy resistance as emergent network properties.

## 1. Introduction

Cancer progression is increasingly recognized as a process driven not only by genetic alterations but also by profound cellular plasticity, enabling tumor cells to dynamically shift between epithelial, mesenchymal, and stem-like states [1,2,3,4]. This plasticity fuels metastasis, therapy resistance, and tumor recurrence, hallmarks that remain major barriers in oncology [5,6]. Recent studies highlight lipid peroxidation as not merely a byproduct of oxidative stress but a decisive modulator of cancer evolution through ferroptosis sensitivity and membrane remodeling, directly linking oxidative metabolism to metastatic behavior.

Clinically, the persistence of these plastic and therapy-resistant tumor states represents a major unmet need, as recent studies reveal that residual “drug-tolerant persister” cells survive even targeted or immune-checkpoint therapies through non-genetic plasticity programs—marked by slow proliferation, metabolic rewiring, and redox-driven epithelial–mesenchymal transitions—leading to inevitable relapse in aggressive cancers such as triple-negative breast cancer, melanoma, and pancreatic ductal adenocarcinoma [7]. Among these processes, epithelial–mesenchymal transition (EMT) and its reverse, mesenchymal–epithelial transition (MET), stand out as pivotal regulators of malignant progression. EMT confers motility and invasiveness through the repression of E-cadherin and the induction of mesenchymal adhesion molecules such as N-cadherin (CDH2), integrins, and AXL receptor tyrosine kinase (AXL) [8]. Strikingly, these features parallel the migratory behavior of neural crest cells during embryogenesis, suggesting a convergence between developmental and oncogenic programs [9,10].

Neural crest stem cells (NCSCs) are transient, multipotent progenitors with extraordinary migratory capacity, giving rise to diverse lineages, including melanocytes, neurons, and craniofacial mesenchyme [8,10]. Reactivation of neural crest-like transcriptional states has been observed in melanoma and other aggressive tumors, where factors such as MSH homeobox 1 (MSX1) and SRY-box transcription factor 10 (SOX10) drive dedifferentiation and enhance invasiveness [9,11,12]. Indeed, melanoma cells can undergo “neural crest-like reprogramming,” marked by a switch from E-cadherin-high to zinc-finger E-box-binding homeobox 1 (ZEB1)-high states, closely mimicking EMT dynamics [9,13]. Similarly, alternative splicing events and transcriptional rewiring reinforce this neural crest–mesenchymal identity, supporting tumor progression and metastasis [14]. These observations collectively argue that the mesenchymal traits acquired through EMT and the developmental logic of the neural crest converge into a shared cellular program that underlies cancer stemness and dissemination.

CSCs, a subpopulation capable of self-renewal and tumor initiation, embody this convergence. CSCs are sustained by a high degree of phenotypic plasticity, enabling interconversion between stem-like and differentiated states in response to microenvironmental cues [5,15]. Such plasticity is orchestrated not only by transcriptional circuits but also by metabolic and signaling adaptations. For instance, CSCs rely heavily on redox balance and metabolic reprogramming, including lipid metabolism, to withstand oxidative stress and maintain stemness [15]. In parallel, exosomes and microRNA (miR) act as key mediators of intercellular communication, propagating EMT and stemness traits across the tumor microenvironment [6,16]. MicroRNA-21, for example, enhances stemness and EMT in pancreatic cancer, reinforcing CSC traits and therapeutic resistance [16,17,18].

Recent single-cell and spatial transcriptomic studies further demonstrate that CSC plasticity is maintained through hybrid epithelial/mesenchymal (E/M) states, where dynamic adhesion remodeling and redox adaptation coexist. These intermediate states provide a survival advantage under oxidative and immune pressure, underscoring the role of redox signaling as a licensing factor for plastic transitions. These hybrid states couple adhesion switching with a mixed-metabolic, nuclear factor erythroid 2-related factor 2 (NRF2)-high program that buffers reactive oxygen species (ROS) and supports persistence under immune pressure [19,20,21,22].

Importantly, these plastic states are often stabilized by tumor microenvironmental signals, including transforming growth factor-β (TGF-β), cytokines, and hypoxia, which sustain EMT transcription factors and redox adaptation [23,24]. Moreover, therapy-induced stress itself may generate new CSC populations via dedifferentiation. For example, chemotherapy can eradicate leucine-rich repeat-containing G-protein-coupled receptor-5-positive (LGR5^+^) CSCs but simultaneously drive the emergence of clusterin-expressing “revival stem cells” that replenish the CSC pool and promote relapse [25].

Similar adaptive programs are reinforced by metabolic and redox reprogramming, enabling survival under oxidative pressure [26]. The tumor immune microenvironment further contributes, as tumor-associated macrophages (TAMs) provide cytokine-rich niches that enhance CSC stemness [27] and immune evasion [28]. Despite the wealth of studies on EMT, neural crest biology, and CSC regulation, a unifying framework that integrates these processes is still lacking. Notably, melanoma and its stromal ecology recapitulate developmental programs: neural crest-like EMT/phenotype switching in melanoma cells, and context-dependent reprogramming of stromal fibroblasts that interact with them. This developmental viewpoint motivates our focus on the under-recognized link between cancer-associated fibroblasts (CAFs) and neural crest-derived lineages [29,30].

Here, we propose that EMT/MET-driven mesenchymal traits and NCSC-like programs converge on a CDH2-centered redox–adhesion–exosome (RAX) hub. This hub coordinates adhesion switches, the redox-buffering NRF2–glutathione peroxidase 4 (GPX4) axis, and exosomal miRNA signaling to stabilize CSC states and promote immune evasion. By positioning redox pulses as the upstream licensing event, we highlight how oxidative stress integrates adhesion remodeling and vesicle-mediated communication into a closed circuit of cancer progression [23,27,31,32]. This perspective not only reconciles developmental and oncogenic paradigms but also identifies actionable vulnerabilities for therapeutic intervention.

## 2. EMT–MET and Neural Crest Convergence

The ability of cancer cells to transition between epithelial and mesenchymal states mirrors developmental processes, particularly those governing neural crest cell migration. EMT and MET endow tumor cells with invasive competence and plasticity reminiscent of NCSCs, suggesting that the molecular logic of embryonic delamination is aberrantly reactivated in tumors. This convergence links adhesion remodeling, transcriptional reprogramming, and migratory potential, establishing a developmental template for tumor progression.

## 3. Neural Crest Stem Cells (NCSCs) and Developmental Plasticity

NCSCs are transient, multipotent progenitors that delaminate from the dorsal neural tube and migrate extensively to form melanocytes, neurons, glia, smooth muscle, and craniofacial mesenchyme [33]. Their EMT-driven delamination parallels carcinoma invasion [34]. Specification is governed by transcriptional networks: paired box 3 (PAX3)/7 and Msx1 activate forkhead box D3 (FoxD3), snail family transcriptional repressor1 (SNAI1), twist family bHLH transcription factor 1 (TWIST1), and SOX10 [35,36,37]. Snail/Slug and Twist repress cadherins, FoxD3 induces SOX10, and SOX10 maintains multipotency [35,38]. Notably, SOX10 alone can reprogram somatic cells into neural crest-like states, underscoring its role as a master regulator of lineage plasticity [35]. Oncogenic EMT exploits the same circuitry. Twist, Snail, and ZEB1—critical in development—are reactivated in tumors to promote invasion and stemness [38]. In melanoma, ZEB1-driven phenotype switching mimics neural crest-like plasticity, while SOX10 re-expression drives dedifferentiation and therapy resistance [39]. Recent single-cell tracing reveals redox-regulated transcriptional nodes (e.g., SOX10–NRF2) stabilizing trajectories under oxidative stress [40]. These parallels highlight a testable hypothesis: developmental EMT circuits are hijacked to sustain cancer stemness and therapeutic resistance, suggesting that tumor plasticity represents an aberrant recapitulation of embryonic logic.

## 4. EMT/MET and Mesenchymal Traits in Cancer

EMT dismantles polarity and adhesion while inducing mesenchymal traits—motility, invasiveness, and resistance to apoptosis [41,42]. MET reverses this process, enabling colonization. EMT is orchestrated by transcription factors (Snail, Twist, ZEB1/2), epigenetic remodeling, and non-coding RNAs, converging on a plastic state highly adaptable and therapy-resistant [42,43]. A defining hallmark is the cadherin switch: loss of CDH1 and induction of CDH2, which destabilizes junctions and promotes extracellular matrix (ECM) engagement and migration [44]. Mesenchymal cells further upregulate integrins (e.g., α5β1), AXL, and platelet-derived growth factor receptor beta (PDGFRB), forming survival circuits [42,45]. Among these, AXL—a TAM family receptor tyrosine kinase—emerges as a key EMT driver. Gas6-mediated AXL activation promotes phosphoinositide 3-kinase (PI3K)/AKT serine/threonine kinase (AKT), mitogen-activated protein kinase (MAPK), and nuclear factor kappa-light-chain-enhancer of activated B cells (NF-κB) signaling, enhancing invasion and therapy resistance [45,46,47]. Elevated AXL correlates with poor prognosis and its inhibition resensitizes tumors [46,47]. Hybrid E/M states identified by single-cell profiling retain partial CDH1 while upregulating CDH2 and integrins. These states exhibit metabolic reprogramming, high NRF2 activity, and PD-L1 expression, allowing persistence under oxidative and immune pressure [19,20]. Thus, EMT is not merely a dissemination mechanism but a licensing step for cancer-stem-cell persistence, linking adhesion remodeling and redox adaptation to immune evasion.

## 5. Convergence Concept

The parallels between EMT and neural crest delamination underscore a biological convergence. Both involve transcriptional reprogramming, cadherin switching, cytoskeletal remodeling, and migratory competence [48]. Single-cell transcriptomics reveal that EMT/MET plasticity generates hybrid E/M states resembling intermediate neural crest stages [49]. These states are marked by partial adhesion remodeling and stemness, bridging embryonic and oncogenic EMT. At the molecular level, cadherin regulation is central. During neural crest EMT, transient downregulation of CDH2 permits delamination, followed by re-expression for collective migration. In cancer, the CDH1-to-CDH2 switch stabilizes mesenchymal adhesion, directional migration, and anoikis resistance [50,51]. CDH2 thus functions not only as an adhesion molecule but as a hub integrating adhesion dynamics, growth factor signaling (AXL, PDGFRB), cytoskeletal polarity, and redox buffering. Emerging evidence shows CDH2 junctions interact with β-catenin, RhoA/Rac1, and antioxidant pathways, enabling cancer stem cells to buffer ROS [50,52]. Spatial single-cell analyses confirm that hybrid states re-express CDH2 in coordination with ROS-buffering circuits, reinforcing plasticity and resilience [53].

Beyond tumor-intrinsic programs, CAFs—including populations arising from non-fibroblast lineages such as endothelial/perivascular cells, adipocytes, monocytes, and bone marrow-derived mesenchymal stem cells (MSC)—amplify RAX circuitry [54]. CAFs are not a single lineage but a state that can arise from multiple sources; critically, adult fibroblasts—including CAFs—retain a positional ‘HOX code’ that reflects their embryonic origin (mesoderm vs. neural crest-derived ectomesenchyme). This developmental imprint persists across inflammatory states and primary/metastatic tumor stroma, implying that a subset of CAFs in head-and-neck/skin contexts plausibly derives from neural crest lineage fibroblasts [55]. By depositing fibronectin/collagens and activating integrin–FAK signaling, CAFs potentiate CDH2/AXL–FAK-dependent adhesion and migration; by releasing paracrine cues and generating redox bursts, they license EMT/NCSC transitions. In addition, cancer cell-derived extracellular vesicle (EV) can drive chemokine programs (e.g., CXCL1/CXCL8), contributing to stromal heterogeneity and worse outcomes [56]. Table 1. summarizes the distinctions from prior EMT/redox/exosome reviews—covering scope, key gaps, and our added contributions (a CDH2-centered closed loop and EV–immune–ferroptosis coupling). Prior work typically treated EMT/CSC plasticity, ferroptosis defenses, exosomal immune circuits, and biomarker strategies as separate silos; none integrated them into a closed-loop model. In contrast, the RAX hub synthesizes adhesion remodeling (CDH2/FAK/AXL), redox buffering (NRF2–GPX4/FSP1–DHODH), and exosomal PD-L1/miRNA circuits into a single interdependent system. These redox-buffering nodes simultaneously regulate lipid peroxidation intensity and ferroptosis threshold, positioning them as biochemical levers of RAX hub homeostasis.

## 6. The CDH2-Centered Redox–Adhesion–Exosome (RAX) Hub

Mounting evidence indicates that cancer progression is driven not by isolated pathways but by integrated networks that stabilize plastic, stem-like, and invasive states. At their intersection lies the cadherin switch, linking adhesion remodeling to redox buffering and exosome-mediated communication. We conceptualize this as the CDH2-centered redox–adhesion–exosome (RAX) hub—a dynamic regulatory node synchronizing adhesion, oxidative adaptation, and intercellular signaling. This section dissects the three axes—adhesion/migration, redox buffering, and exosome-mediated communication—and integrates them into a closed feedback circuit.

## 7. Adhesion and Migration Axis

The cadherin switch, defined by loss of CDH1 and induction of CDH2, dismantles epithelial junctions and establishes mesenchymal adhesions, enhancing motility and invasion [95,96]. CDH2 supports stable collective migration and couple cells to extracellular matrix (ECM) via integrins [96,97]. Integrin–FAK/Src cascades remodel actin cytoskeleton, drive directional motility, and confer anoikis resistance [98,99]. Mesenchymal stability depends on a CDH2–AXL–PDGFRB triad. AXL activation via Gas6 triggers PI3K/AKT, MAPK, and NF-κB signaling to reinforce invasion and drug resistance [99,100], while PDGFRB amplifies PI3K–MAPK pathways and vascular integration [97,101]. Together, this triad forms a positive feedback loop: CDH2 facilitates AXL clustering, AXL enhances mesenchymal gene expression, and PDGFRB stabilizes survival signaling. Hybrid E/M states, defined by partial CDH1 retention with CDH2 and integrin upregulation, display enhanced metastatic efficiency and adaptability [96]. Importantly, tumor-derived exosomes propagate this circuitry. For example, TNBC cells export integrin β4 (ITGB4) to fibroblasts, inducing mitophagy and glycolysis that metabolically prime stroma for invasion; ITGB4 knockdown or exosome blockade disrupts this process [102]. Conversely, exosomal DOC2B can elevate ROS and lipid peroxidation, sensitizing cells to chemotherapy [103]. These findings support the perspective that exosomes act not as byproducts but as context-specific editors of adhesion and migration.

## 8. Redox-Buffering Axis

Reactive oxygen species (ROS) act both as signaling intermediates and cytotoxic stressors. In cancer, transient ROS pulses license EMT and NCSC-like transitions by coupling to cadherin switching and transcriptional reprogramming [104]. Intravital imaging reveals localized H_2_O_2_ surges at invasive fronts temporally aligned with EMT onset [105,106]. To survive oxidative bursts, CSC-like cells activate antioxidant defenses. The NRF2–Kelch-like ECH-associated protein 1 (KEAP1) axis regulates genes including Solute carrier family 7 member 11 (SLC7A11), Glutamate–cysteine ligase catalytic subunit (GCLC), NAD(P)H quinone dehydrogenase 1 (NQO1), heme oxygenase-1 (HO-1), and GPX4 [107,108], thereby sustaining cystine uptake, glutathione (GSH) synthesis, and peroxide detoxification. The GSH–GPX4 circuit reduces phospholipid hydroperoxides and blocks ferroptosis [108,109], while ACSL4 and epigenetic GPX4 regulation fine-tune ferroptosis sensitivity [109,110]. Parallel modules—FSP1-driven CoQ10 and DHODH-driven CoQH2—further trap lipid radicals [111]. These buffering systems stabilize EMT/CSC states but also expose a therapeutic vulnerability: mesenchymal-like cells resist apoptosis yet remain selectively sensitive to ferroptosis triggers [104]. From a perspective standpoint, this paradox defines a new Achilles’ heel—disrupting NRF2–GPX4–FSP1/DHODH axis defenses may collapse redox tolerance and eliminate therapy-resistant CSC pools.

## 9. Exosome–miRNA Axis

Exosomes link oxidative stress to adhesion/migration programs. Their secretion involves ESCRT complexes and RAB GTPases (RAB27A/B) [112], with stress pathways such as p53–TSAP6 further enhancing release [113]. Redox stress not only increases vesicle output but also dictates selective cargo loading [114]. The miR-200 family regulates EMT plasticity by suppressing ZEB1/2, while paradoxically aiding metastatic seeding [115]. miR-21 and miR-210 are enriched in hypoxic/redox-stressed exosomes, driving glycolysis, HIF stabilization, and immune evasion [112,116]. Cargo selection is actively controlled: YBX1 directs miR-223 loading, hnRNPA2B1 recognizes EXO-motifs under SUMOylation, [105] and ESCRT adaptors ALIX/TSG101 govern membrane scission and export [117]. Within the RAX hub, these selectors bias cargo toward miR-21/miR-210 and PD-L1 under oxidative stress, amplifying redox–adhesion programs [118]. Oxidative stress—including lipid–aldehyde stress—increases EV release, while hypoxia/ROS signaling elevates exosomal PD-L1 and reshapes immunosuppression; EV miR-21/210 are enriched under redox stress, linking lipid peroxidation to immune checkpoint up-regulation and EMT/CSC support [119]. Lipid peroxidation is initiated by the oxidation of polyunsaturated fatty acids (PUFAs), producing lipid radicals (L•) that propagate chain reactions to form lipid hydroperoxides (LOOH). The subsequent decomposition of unstable LOOH species generates reactive aldehydes, mainly 4-hydroxynonenal (4-HNE) and malondialdehyde (MDA). Recent studies have demonstrated that elevated 4-HNE derived from n-6 PUFA metabolism amplifies oxidative stress-driven signaling cascades such as ASK1–JNK and suppresses AMPK activation, thereby linking lipid peroxidation to redox-dependent transcriptional reprogramming [120]. These electrophilic aldehydes form Michael adducts with cysteine, histidine, and lysine residues on key regulators of epithelial–mesenchymal transition (EMT) and cytoskeletal remodeling, including NF-κB and Nrf2, modulating gene expression associated with cell adhesion and motility. Moreover, long-lived species that maintain lower PUFA content and reduced 4-HNE/MDA burden exhibit enhanced glutathione-S-transferase (GST) activity and resilience to free-radical stress [121], highlighting 4-HNE as a pivotal mediator coupling membrane lipid composition to redox homeostasis. Through this mechanism, lipid–aldehyde stress may directly influence selective exosomal cargo loading—favoring miR-21 and miR-210 packaging—and thereby reinforce EMT plasticity and immune evasion within the RAX hub. Exosomal cargo thus functions as a mobile amplifier. In melanoma, PD-L1^+^ or FasL^+^ vesicles impair T cell cytotoxicity and condition pre-metastatic niches [116]. Therapy-induced exosomes further export resistance-promoting miRNAs [74]. Exosomes are active network players, reinforcing malignant states across stromal, vascular, and immune compartments, not passive waste vesicles.

## 10. Integration as a Closed Circuit

**Figure 1:**
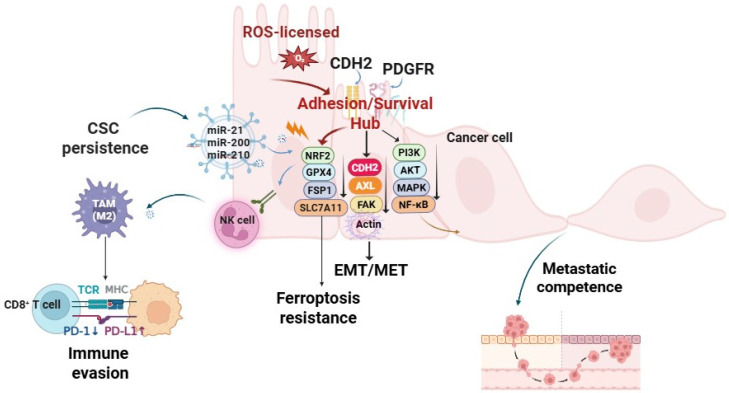
Transient reactive oxygen species (ROS) pulses are stabilized into persistent malignant states through sequential licensing, lock-in, redox buffering, and broadcasting phases. Licensing: ROS-driven epithelial–mesenchymal transition (EMT) initiates cadherin switching from E-cadherin to N-cadherin (CDH2). Lock-in: CDH2 cooperates with integrins and receptor tyrosine kinases (AXL, PDGFRB) to activate FAK/Src–PI3K/AKT–MAPK signaling, reinforcing adhesion, migration, and mesenchymal stability. Redox buffering: NRF2, GPX4, FSP1, and SLC7A11 restore lipid redox homeostasis and confer ferroptosis-resistance. Broadcasting: selective exosomal cargo—miR-21, miR-200, miR-210, and PD-L1—propagate stemness, remodel the tumor stroma, and mediate immune evasion. Collectively, this closed-loop RAX circuit integrates oxidative signaling, adhesion dynamics, and exosome-mediated communication to sustain cancer cell plasticity and therapy resistance. Arrows indicate direction of regulation: ↓ downregulation, ↑ upregulation. Abbreviations: AKT: protein kinase B; AXL: AXL receptor tyrosine kinase; CDH2: N-cadherin; CSC: cancer stem cell; EMT/MET: epithelial–mesenchymal transition/mesenchymal–epithelial transition; FAK: focal adhesion kinase; FSP1: ferroptosis suppressor protein 1; GPX4: glutathione peroxidase 4; MAPK: mitogen-activated protein kinase; MHC-I: major histocompatibility complex class I; NF-κB: nuclear factor kappa-light-chain-enhancer of activated B cells; NRF2: nuclear factor erythroid 2-related factor 2; PD-1: programmed cell death protein 1; PD-L1: programmed death-ligand 1; PDGFRB: platelet-derived growth factor receptor beta; SLC7A11: solute carrier family 7 member 11; PI3K: phosphoinositide 3-kinase; TAM: tumor-associated macrophage; TCR: T cell receptor. Created in BioRender. Kim, B. (2025) (License number: YS292KS8UO).

The RAX hub functions as a self-reinforcing feedback loop in which redox, adhesion, and exosomal pathways converge to sustain cellular plasticity. Transient ROS pulses activate EMT and NCSC transcription factors, thereby initiating the cadherin switch from CDH1-to-CDH2 [122]. Subsequent activation of integrin–FAK/Src signaling amplifies migratory and survival programs, consolidating the mesenchymal phenotype. In parallel, EMT reprogramming skews exosomal cargo toward mesenchymal and redox-regulatory microRNAs—notably miR-200, miR-21, and miR-210 [123,124], which propagate cancer stem cell (CSC) traits and reinforce immune suppression. These processes are further stabilized by antioxidant defense modules, including NRF2, GPX4, FSP1, and DHODH [125], which buffer oxidative pressure and preserve redox homeostasis. Together, the CDH2-centered RAX hub integrates adhesion, redox, and exosomal axes into a self-reinforcing circuit that converts transient oxidative cues into durable malignant identities—licensing EMT/NCSC transitions, stabilizing CSC pools, and sustaining immune-evasive, therapy-resistant states. Exosomal PD-L1 and immunosuppressive miRNAs blunt CD8^+^ T cell activity and promote Treg expansion [79,126]. Therapeutically, concurrent targeting of adhesion (CDH2/AXL–FAK), redox buffering (GPX4/SLC7A11, FSP1, DHODH), and exosomal signaling offers a rational strategy to disrupt this circuit (Figure 1).

## 11. Functional Outcomes of the RAX Hub

The integration of adhesion, redox, and exosome–miRNA signaling within the CDH2-centered RAX hub converges on three malignant hallmarks: maintenance of cancer stemness, metastatic competence, and immune evasion [127,128].

## 12. Cancer Stemness and Plasticity

Transient ROS pulses license E/M switching and NCSC programs. This plasticity is stabilized by CDH2–AXL/FAK signaling and insulated from ferroptosis by nuclear factor erythroid 2-related factor 2 (NRF2)-driven SLC7A11/GPX4 and the ferroptosis suppressor protein 1 (FSP1) axis. Concomitantly, hypoxia/redox stress biases exosomal cargo toward miR-200/210 and programmed death-ligand 1 (PD-L1), exporting plasticity and suppressing cytotoxic immunity [129]. Operational readouts include tumorsphere-formation efficiency, aldehyde dehydrogenase (ALDH) activity, CD44^high/CD24^low fractions, and exosomal PD-L1/miR-200/210 abundance [129,130]. This hybrid circuitry enables dynamic switching between quiescent and invasive states, preserving tumor growth potential [131]. Phenotype switching in melanoma and neuroblastoma illustrates this principle: cells toggle between MITF^high proliferative and MITF^low invasive states, mirroring CSC dormancy versus migratory phenotypes [128,131]. Exosomes reinforce stemness by horizontally transferring miRNAs (miR-21, miR-210) and stemness regulators to non-CSCs, expanding the CSC pool beyond original clones [106,126]. Redox buffering via NRF2 stabilization, GPX4-dependent lipid detoxification, and FSP1-mediated ferroptosis-resistance protects CSCs against ROS surges during EMT/NCSC transitions [126]. Single-cell and spatial transcriptomics confirm that hybrid E/M states are persistent, niche-anchored programs enriched for CSC traits and paracrine signaling [132]. In neuroblastoma, longitudinal profiling identifies cycling between adrenergic and mesenchymal-like states with a discrete “bridge” population, echoing neural crest plasticity [105]. Collectively, these data nominate CSC plasticity as a key outcome of the RAX hub and highlight therapeutic opportunities in ferroptosis sensitization and exosome disruption.

## 13. Metastatic Competence

The CDH2/integrin/AXL axis operates as a motility engine: CDH1 loss and CDH2 induction destabilize junctions, while integrin β1/β3–ECM binding activates FAK/Src cascades for migration [133,134]. AXL activation further stabilizes mesenchymal states and correlates with poor prognosis [134]. Tumor-derived exosomes extend this program by priming pre-metastatic niches. Integrin-defined exosomal tropism (α6β4/α6β1 for lung; αvβ5 for liver) remodels ECM and recruits stromal/immune cells, generating permissive colonization sites [135]. Exosomal gangliosides and CAMs activate stromal PDGFRB–FAK–ERK signaling, amplifying metastatic support [136]. Thus, intrinsic adhesion dynamics and extrinsic vesicle conditioning jointly fuel metastatic competence. Hybrid E/M states enriched at invasive edges exemplify this coupling.

## 14. Immune Evasion

The RAX hub subverts immune surveillance through vesicle-mediated signaling. Exosomal PD-L1 acts as a decoy for PD-1, impairing cytotoxic T lymphocytes; PD-L1^+ vesicles are enriched in glioblastoma, HCC, and GI cancers, correlating with checkpoint resistance [137,138]. Vesicular miRNAs (miR-21, miR-146a, miR-210) reprogram macrophages and dendritic cells into tolerogenic states, expanding FOXP3^+^ Tregs [139,140]. Exosome-conditioned TAMs adopt M2-like phenotypes, secreting IL-10/TGF-β and recruiting Tregs via CXCL12–CXCR4 signaling [141,142]. Collectively, immune evasion emerges as a structurally integrated outcome of the RAX hub, explaining why PD-L1 blockade alone is insufficient and why vesicle biogenesis/cargo inhibition must be combined with checkpoint therapies [143,144].

## 15. Therapeutic Targeting of the RAX Hub

Because the RAX hub integrates EMT plasticity, CSC persistence, and immune evasion, targeting its nodes offers the possibility of collapsing multiple malignant programs simultaneously [140,145].

## 16. Adhesion/Signaling Inhibition

Anti-N-cadherin antibodies, notably the monoclonal antibody ADH-1 (Exherin), disrupt CDH2-mediated adhesion and attenuate integrin–FAK/Src signaling, thereby reducing invasion and enhancing chemosensitivity [146]. Focal adhesion kinase (FAK) inhibitors, including Defactinib and GSK2256098, decrease motility, disrupt CSC niches, and attenuate CAF signaling; importantly, they also diminish exosome release [147]. AXL blockade, represented by small-molecule inhibitors (bemcentinib/BGB324, cabozantinib) as well as therapeutic antibodies, interferes with Gas6–AXL signaling, reverses EMT traits, and restores sensitivity to tyrosine kinase inhibitors (TKIs) and checkpoint blockade [148]. Collectively, inhibition of the CDH2–integrin–AXL–FAK axis illustrates how adhesion disruption destabilizes the RAX hub.

## 17. Redox Vulnerabilities

Covalent GPX4 inhibitors, including RSL3, ML162, and the next-generation compound C18 [149,150], induce ferroptosis in triple-negative breast cancer (TNBC) models [150,151,152]. Agents such as sulfasalazine and erastin deplete cysteine, diminish glutathione (GSH) pools, and trigger ferroptotic death [153]. Dual blockade of GPX4 and the cystine transporter xCT collapses antioxidant defenses and amplifies susceptibility to ferroptosis. Parallel pathways also contribute: FSP1 regenerates ubiquinol to trap lipid radicals, while inhibition of DHODH with brequinar sensitizes GPX4^high tumors, particularly when combined with xCT blockade [153]. Additional pharmacological interventions, including statins, withaferin A, or radiotherapy, intensify lipid peroxidation and synergize with GPX4 or xCT inhibition to overwhelm CSC defenses [149,154]. EMT-associated upregulation of ACSL4 and LPCAT3 promotes PUFA enrichment in membranes, enhancing metastatic adaptability but simultaneously sensitizing cells to ferroptotic stress under GPX4 inhibition. Phosphorylation of ACSL4 by PKCβII and PCK2 further amplifies ferroptotic signaling by increasing its phospholipid remodeling capacity. Conversely, induction of monounsaturated lipid synthesis via SCD1 or LPCAT1 confers ferroptosis resistance in epithelial-like cancer cells [155]. Thus, ferroptosis resistance in EMT/CSC populations represents a dynamic equilibrium between antioxidant buffering (NRF2–GPX4/FSP1/DHODH) and pro-oxidant remodeling (ACSL4–PUFA axis), rather than a fixed phenotype [156].

## 18. Exosome-Targeting Approaches

Targeting exosomal pathways provides complementary vulnerabilities. Inhibition of Rab27a or ESCRT machinery reduces the export of oncogenic cargo [157]. AntagomiRs or RNA sponges can neutralize oncogenic miRNAs such as miR-21, miR-210, and the miR-200 family, while engineered scaffolds have been developed to sequester these RNAs [158,159]. Moreover, blockade of RNA-binding protein transfer prevents CSC stabilization and therapy resistance [158]. Importantly, combining exosome targeting with ferroptosis inducers or immune checkpoint inhibitors may yield synergistic anti-tumor effects.

## 19. Multi-Node Targeting Strategy

Preclinical studies indicate that single-axis inhibition can be bypassed by compensatory circuits across adhesion (CDH2/AXL–FAK), redox defenses (GPX4–SLC7A11; ferroptosis-modulating FSP1/DHODH), and exosomal programs (EV cargo/PD-L1). These observations support evaluating multi-node combinations in selected contexts, rather than establishing a universal requirement [159,160]. For example, dual inhibition of GPX4 and xCT enhances ferroptotic cell death in colorectal and ovarian cancer models [161], while suppression of exosome pathways has reduced drug resistance [159,162]. Adhesion blockade through anti-CDH2 antibodies or AXL/FAK inhibitors further limits dissemination and primes tumors for redox collapse [163]. Thus, combining CDH2 interference, GPX4 inhibition, and EV pathway suppression may create synthetic lethality-like vulnerabilities in selected preclinical contexts, warranting prospective testing with endpoints and assay standardization [164]. Nanotechnology and engineered vesicles offer translational platforms to implement such multi-node strategies [163]. CAF ontogeny/positional-code PD: regional HOX gene panels (e.g., HOXA9/HOXD9, MEIS1/PRDM6) in CAF clusters, aligned with neural crest-enriched tumor states and myeloid-chemokine programs [55]. As operational exemplars, Table 2 summarizes axis-pairing combinations with matched PD readouts and early-phase design considerations.

## 20. Clinical Translation: Targeting Adhesion, Redox, and Exosomal Axes

Emerging clinical trials underscore the translational feasibility of dismantling the RAX hub through multi-node interventions. Adhesion signaling: AXL inhibitors such as bemcentinib (NCT02424617, NCT03184571, NCT03649321) and FAK inhibitors such as defactinib (NCT02758587, NCT03727880) are under evaluation for their ability to suppress EMT-driven dissemination and synergize with immune checkpoint blockade [62,171]. Redox buffering: Pharmacologic induction of ferroptosis through GPX4 inhibitors (e.g., FIN56 analog, NCT04205357) and IKE derivatives (NCT04836577) highlights ferroptosis defense as a tractable therapeutic vulnerability in solid tumors [172,173]. Exosomal circuits: In parallel, strategies targeting exosome secretion or employing engineered exosome carriers are advancing, including plant-derived nanovesicles (NCT03608631) and KRAS siRNA-loaded exosomes (NCT04747574) [174]. Together, these trials reinforce the clinical relevance of targeting adhesion (CDH2/AXL–FAK), redox buffering (GPX4/SLC7A11; FSP1), and exosomal signaling (PD-L1/miRNAs) as a biomarker-guided therapeutic triad to disrupt the malignant RAX hub. Clinically, developmental readouts could be co-opted as stromal stratifiers: positional HOX-code-based annotation of CAF compartments in head/neck and cutaneous tumors, and spatial co-profiling of neural crest-like tumor states with CAF activation and myeloid recruitment. While hypothesis-generating, these proposals are mechanistically grounded in developmental programs observed in human fibroblasts and in vivo melanoma models [55,175]. To contextualize the RAX hub in human tumors, we re-analyzed TCGA and published cohorts. Elevated CDH2 and miR-210 consistently associated with poor survival across colorectal, lung, breast, and PDAC datasets. Notably, patients with CDH2^high/miR-210^high tumors exhibited the worst prognosis (Table 3). These findings reinforce the clinical relevance of the RAX framework and support its evaluation as a prognostic–predictive tool in biomarker-enriched trials.

## 21. Implications and Future Perspectives

The CDH2-centered RAX hub (redox–adhesion–exosome network) provides tractable axes for diagnosis, patient stratification, and therapy monitoring. Liquid-biopsy-ready exosomal readouts, when coupled with dynamic EMT/NCSC state sensors, could enable personalized disruption of the hub. This view is consistent with recent evidence highlighting biomarkers as central to diagnosis, therapeutic decision-making, and longitudinal monitoring in oncology [181]. In this respect, the RAX framework resonates with the recently proposed ‘ADAPT’ paradigm, advocating feedback-driven, adaptive strategies to counter cancer evolution [182].

## 22. Biomarkers: Exosomal CDH2/miR-200/miR-210 Panel

Exosomes are stable carriers of proteins and non-coding RNAs (ncRNAs) in body fluids such as blood, urine, and cerebrospinal fluid, making them highly suitable as non-invasive cancer biomarkers and theranostic tools. Their cargo reflects the dynamic state of the tumor and changes with disease progression [183]. Within this framework, CDH2 protein represents the adhesion and migration arm; exosomal proteomes enriched for adhesion molecules stratify metastatic tropism, and the inclusion of N-cadherin constitutes a testable extension [184]. The miR-200 family functions as a gauge of plasticity and EMT state, since the ZEB1–miR-200 axis governs the EMT/MET balance, and miR-200-based sensors are already employed as live reporters of EMT dynamics [106]. In parallel, miR-210 acts as a sentinel for hypoxia and redox stress, linking hypoxic conditions to EMT-to-NCSC transitions and CSC stabilization, with validation in lung and pancreatic models [185]. Clinical precedent strongly supports this biomarker concept: exosomal proteins including TYRP2, VLA-4, MET, and caveolin-1 predict outcomes in melanoma, while exosomal miRNAs such as miR-21 have been shown to track therapy resistance in lung cancer [112,183]. A practical validation path involves discovery in banked plasma or urine samples using targeted exosome isolation and orthogonal quantification (ELISA or PRM for CDH2, RT-qPCR for miRNAs), followed by the definition of cut-off thresholds in prospective cohorts. Importantly, urine- and plasma-based exosomal panels already distinguish cancer states and monitor therapeutic responses, underscoring the translational feasibility of this approach [183]. Similar integrative strategies combining TCGA datasets with survival data have successfully identified robust progression gene signatures, supporting the feasibility of the RAX biomarker panel [186].

## 23. Patient Stratification: EMT-High/NCSC-High Tumors as “RAX-Vulnerable”

Tumors with pronounced EMT plasticity and neural crest-like transcriptional programs are predicted to be most dependent on the RAX circuitry and therefore most susceptible to its disruption. In melanoma, CD271 and SOX10 identify highly plastic, therapy-resistant, and metastasis-prone states, making this tumor type an archetype of RAX dependence [39,187]. In neuroblastoma, co-expression of SOX10 and PHOX2B alongside canonical CSC markers such as CD133 and ALDH1 delineates an NCSC-like program that is associated with aggressiveness and poor survival [187]. Lung cancers with hypoxia-driven plasticity also represent promising candidates: exosomal miR-210 derived from lung CSCs promotes metastasis, while neuroendocrine-lineage lung tumors, including subtypes of small-cell lung cancer (SCLC), may likewise be considered for RAX-targeted interventions, although tumor type-specific validation remains essential [185]. Operationally, tissue immunophenotyping (CDH2 ↑/CDH1 ↓; SOX10/CD271 where relevant) should be paired with the liquid-biopsy RAX panel to guide assignment into RAX-targeted combinations.

## 24. Future Direction: Toward Personalized Therapy by Disrupting the RAX Hub

Future translational strategies should prioritize dynamic state tracking rather than static lineage assessment. The use of Z-cad-style EMT/MET sensors in patient-derived organoids and preclinical models can enable real-time monitoring of plasticity under drug pressure, thereby improving relapse prediction and guiding the rational sequencing of therapies [106]. Complementary liquid-biopsy monitoring, based on serial quantification of exosomal CDH2, miR-200, and miR-210, offers a minimally invasive means of detecting RAX rebound or hub collapse. Circulating miRNAs have already demonstrated feasibility by correlating with tumor burden and normalizing post-resection in multiple cancer contexts [188,189]. An immediate translational implication is that stromal composition and fibroblast ontogeny are not neutral: macroH2A loss in an autochthonous melanoma model increases CAF frequency/activation and promotes neural crest-lineage dedifferentiation with immunosuppressive myeloid influx—features linked to impaired anti-tumor immunity [175]. Moving forward, clinical trial designs should selectively enrich for EMT-high and NCSC-high tumors such as melanoma and neuroblastoma, where RAX dependence is most pronounced. Multi-node therapeutic combinations—targeting the CDH2/AXL–FAK adhesion axis together with ferroptosis vulnerabilities (GPX4/xCT) and exosome biogenesis—can be deployed within biomarker-guided escalation and de-escalation frameworks, as outlined in the therapeutic targeting and clinical translation sections. This approach provides a roadmap for implementing plasticity-aware precision oncology that integrates mechanistic insight with personalized therapy design. Future extensions could integrate AI-enabled mechanistic models of tumor growth, enabling biomarker-enriched personalization of RAX-targeted interventions [190].

## 25. Discussion

The RAX hub framework offers a mechanistic synthesis of redox signaling, adhesion dynamics, and exosomal communication, and may serve as a conceptual blueprint for precision oncology. From a diagnostic perspective, exosomal cargo—including adhesion molecules such as CDH2, regulatory miRNAs like the miR-200 family and miR-210, together with immune checkpoint proteins such as PD-L1—represents a promising source of liquid-biopsy biomarkers. These vesicle-derived signatures not only reflect EMT status, hypoxic adaptation, and therapy resistance but also provide minimally invasive tools for monitoring tumor plasticity and predicting patient response to immune checkpoint blockade [191,192].

Lipid peroxidation generates reactive aldehydes such as 4-HNE and MDA, which covalently modify cysteine, histidine, or lysine residues in membrane and cytosolic proteins, forming stable Michael adducts that alter adhesion dynamics and transcriptional control. 4-HNE accumulates at the invasive front of tumors, where it promotes E-to-N-cadherin switching and FAK/Src activation, driving EMT and CAF reprogramming through NF-κB signaling [193,194]. Through Keap1 adduction and GSK3β inhibition, 4-HNE activates Nrf2 and stabilizes mesenchymal persistence within the RAX hub [195]. Conversely, MDA-derived adducts serve as neoantigenic determinants that induce T cell exhaustion and immune tolerance, further reinforcing redox-driven immune evasion [196]. Clinically, plasma levels of 4-HNE and MDA correlate with tumor stage and oxidative burden, suggesting their potential utility as liquid-biopsy biomarkers of lipid peroxidation intensity and RAX hub activity [197]. Moreover, oxidative stress enhances 4-HNE-dependent exosomal export of PD-L1 and miR-21/210, providing a molecular bridge between lipid peroxidation and immune escape pathways [198].

These biomarkers are measurable in plasma and urine, offering real-time assessment of cancer stemness, therapy resistance, and metastatic risk. While CAFs are widely recognized by clinicians, their developmental provenance is often overlooked; converging evidence indicates that CAF states interweave with neural crest biology—both via persistent positional codes in adult fibroblasts and via tumor-driven re-engagement of neural crest-like programs in melanoma [29,55]. Rather than proposing a new lineage origin, this emerging concept highlights that fibroblast plasticity and neural crest-linked transcriptional memory may share a conserved developmental logic. This developmental parallel may help explain the remarkable heterogeneity, migratory capacity, and therapy resistance of CAF, emphasizing the importance of re-examining stromal biology through a neural crest-inspired lens. By contextualizing CAF behavior within broader principles of developmental reprogramming, this framework aligns with established literature yet opens a safer, mechanistically grounded path for understanding tumor–stroma evolution.

On the therapeutic side, the closed-circuit nature of the RAX hub underscores the necessity for multi-pronged interventions. Concurrent inhibition of adhesion-driven plasticity (CDH2 and AXL–FAK signaling) [109], ferroptosis-resistance circuits (the GPX4–xCT antioxidant axis and the FSP1–DHODH mitochondrial pathway) [199,200,201], and exosome biogenesis or selective cargo loading (Rab27a, ALIX, YBX1) could synergistically dismantle the adaptive resilience of cancer stem-like cells [200]. Through covalent Keap1 modification and GSK3β phosphorylation, 4-HNE creates a feed-forward Nrf2 loop that reinforces the RAX-dependent ferroptosis-defense network [195]. Such integrated targeting strategies may disrupt both metabolic and EV-mediated defenses, thereby enhancing the efficacy of conventional and immunotherapeutic modalities. This strategy provides a rational framework for designing multi-node therapies that could overcome resistance to immune checkpoint blockade, ferroptosis inducers, and targeted agents.

Clinically, tumors enriched in EMT and NCSC-like signatures—including melanoma, neuroblastoma, small-cell lung cancer (SCLC), and defined subsets of pancreatic ductal adenocarcinoma (PDAC) and triple-negative breast cancer (TNBC)—represent high-priority candidates for biomarker-driven clinical trials. In patients, plasma MDA (or MDA-protein adducts) levels strongly correlate with tumor stage and oxidative load, validating its potential as a circulating redox biomarker [202]. Elevated MDA-protein adducts in patient plasma positively correlate with oxidative load and advanced tumor stage [197]. Such tumors display pronounced cellular plasticity, therapeutic resistance, and immune-evasive traits, underscoring the translational potential of stratifying patients on the basis of EMT/NCSC transcriptional program [203,204,205]. Incorporating RAX hub signatures into patient stratification could refine therapeutic decision-making and identify subsets most likely to benefit from plasticity-aware interventions. In neuroblastoma, stromal neural crest progenitors exhibit marked radioresistance, undergo α-SMA-positive CAF-like differentiation after irradiation, and support regrowth of tumor neuroblasts—illustrating how neural crest-derived stroma can shape treatment response in vivo [206]. Collectively, these data argue that parts of the CAF compartment are developmentally imprinted and, in head–neck/skin settings, plausibly neural crest-derived; in melanoma, stromal–tumor crosstalk further re-engages neural crest programs with consequences for immunity and therapy response [55,175].

Ultimately, the convergence of liquid-biopsy biomarkers—including circulating tumor cells, cfDNA/ctDNA, and EV—with state-aware preclinical models such as organoids and patient-derived xenografts (PDXs) provides a robust translational framework. These platforms not only preserve tumor heterogeneity and therapeutic vulnerabilities but also enable parallel testing of rational drug combinations, thereby establishing a roadmap that bridges biomarker discovery with precision clinical intervention [207,208,209]. This approach may bridge the gap between mechanistic insight and clinical application, positioning the RAX hub as an actionable target in next-generation oncology. Integrating spatial transcriptomics and exosomal miRNA profiling would further strengthen mechanistic insight into redox-driven plasticity and intercellular communication. Recent studies utilizing single-cell and spatially resolved technologies have revealed how tumor-derived exosomes reprogram immune cells through specific miRNA cargo, such as miR-424 (322)/503, miR-21, and miR-155, thereby reinforcing therapy resistance and niche remodeling in cancer stem cell populations [94,210,211]. Oxidative–aldehyde stress enhances exosomal PD-L1 and miR-21/210 release, linking lipid peroxidation to immune checkpoint up-regulation [198]. Particularly, spatial transcriptomics combined with exosome-tracking approaches [211] enable precise localization of exosome–immune interactions in the tumor microenvironment, which could be applied to validate CDH2-centered redox licensing and EMT–CSC coupling. Furthermore, profiling of exosomal miRNAs from hypoxia- or ROS-conditioned cancer cells has provided functional evidence of redox-regulated vesicle content reprogramming [94,210,211].

## 26. Positioning and Strengths

**Figure 2:**
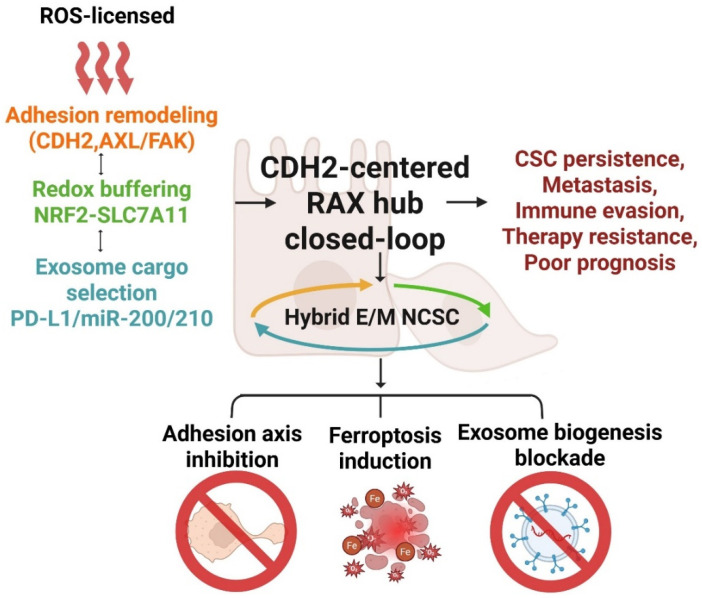
Schematic model of the CDH2-centered redox–adhesion–exosome (RAX) hub illustrating its closed-loop architecture in cancer stemness and therapy resistance. Transient reactive oxygen species (ROS) pulses trigger adhesion remodeling through coordinated activation of CDH2, AXL, and FAK, which in turn couple with NRF2–SLC7A11-driven redox buffering and selective exosomal cargo enrichment (PD-L1, miR-200/210). These interlinked mechanisms form a self-reinforcing circuit that maintains hybrid epithelial/mesenchymal (E/M) and neural crest stem-like (NCSC) states, thereby promoting cancer-stem-cell (CSC) persistence, metastasis, immune evasion, and therapeutic resistance. The bottom panel highlights three therapeutic avenues—adhesion axis inhibition, ferroptosis induction, and exosome biogenesis blockade—as potential interventions to disrupt this adaptive plasticity network. Abbreviations: AXL: AXL receptor tyrosine kinase; CDH2: Cadherin-2; FAK: focal adhesion kinase; NRF2: nuclear factor erythroid 2–related factor 2; SLC7A11: solute carrier family 7 member 11; PD-L1: programmed death-ligand 1; E/M: epithelial/mesenchymal; NCSC: neural crest stem cell; CSC: cancer stem cell; ROS: reactive oxygen species. Created in BioRender. Kim, B. (2025) (License number: SU291KFI04).

The CDH2-centered RAX hub provides an integrative framework unifying three previously disconnected axes of cancer biology: redox dynamics, adhesion signaling, and exosome-mediated communication. Whereas EMT, CSC, and EV studies have historically advanced in parallel, this model synthesizes them into a closed circuit in which transient ROS pulses license EMT/NCSC transitions, CDH2 switching anchors mesenchymal persistence, and selective exosomal cargo propagates these programs across the tumor microenvironment. A key strength is explanatory breadth. Intravital imaging and HyPer probe studies demonstrate that EMT is punctuated by localized ROS bursts coinciding with N-cadherin upregulation and invasiveness, validating ROS pulses as in vivo “licensing signals” [212]. On the vesicular side, biochemical genetics shows that exosomal cargo loading is actively regulated by RNA-binding proteins (YBX1, hnRNPA2B1) and ESCRT adaptors (ALIX, TSG101), with functional consequences for migration and invasion [213]. Single-cell and spatial transcriptomics further confirm that hybrid E/M states are stable, niche-organized programs enriched for CSC markers and paracrine signaling [214]. Together, these converging data support the RAX hub as a multi-axis driver of cancer stemness, invasion, and resistance. This multi-axis interaction is schematized, illustrating how ROS-licensed CDH2 switching, redox buffering, and exosomal cargo selection are integrated into a self-reinforcing RAX hub driving hybrid E/M and NCSC programs (Figure 2). 4-HNE-induced FAK phosphorylation and cytoskeletal remodeling further sustain mesenchymal motility within the adhesion arm of the RAX hub [193,194]. Through covalent KEAP1 cysteine adduction and Akt-dependent GSK3β Ser9 phosphorylation, 4-HNE promotes NRF2 stabilization and a feed-forward antioxidant loop that reinforces the RAX-dependent ferroptosis–defense network [196,198].

## 27. Limitations

Despite converging evidence, the framework remains largely preclinical. ROS pulsing has been studied mainly with live-cell probes in xenografts and organoids, leaving spatiotemporal dynamics in patient tumors unresolved. Similarly, while YBX1/hnRNPA2B1/ALIX are validated cargo selectors in vitro, there in vivo role in shaping metastatic niches is not fully proven. Single-cell EMT trajectories robustly identify hybrid states, but caveats remain—batch effects, sequencing depth, and trajectory inference may distort their prevalence across datasets [215]. Moreover, most evidence derives from neural crest-derived tumors (melanoma, neuroblastoma, SCLC); generalizability to pancreatic, colorectal, and breast cancers requires further validation.

Furthermore, tumor-type heterogeneity must be acknowledged. While the RAX circuitry captures core features of redox–adhesion–exosome crosstalk, EMT and adhesion programs vary across malignancies, and certain tumors exhibit mesenchymal plasticity independent of N-cadherin or canonical EMT markers. Likewise, technical inconsistencies in EV isolation and characterization—ranging from ultracentrifugation to polymer-based precipitation—can confound comparative analyses of exosomal PD-L1 or miRNA content between studies. Standardization of EV handling and inclusion of multiple adhesion markers (e.g., vimentin, integrin-β1) will be necessary to ensure reproducibility and translational relevance.

Three mechanistic gaps deserve focused resolution: First, causal imaging of ROS pulses, EMT entry, and CDH1-to-CDH2 switching in the same cells in vivo. Second, genetic validation that YBX1/hnRNPA2B1/ALIX govern EV cargo choice and control metastasis/immune tone. Third, harmonized atlases quantifying EMT state transitions and exosomal PD-L1/miR-210 signals in patient tissues across disease stages. There is a clear need for integrated single-cell, spatial, and liquid-biopsy validation. To mitigate the risk of overextension, we propose a staged validation roadmap with pre-specified Go/No-Go thresholds. In the preclinical tier, ≥2 pharmacodynamic (PD) axes (e.g., CDH2–FAK phosphorylation decrease; lipid ROS increase; EV-PD-L1 suppression) must be activated without dose-limiting toxicity to proceed. In the translational tier, biomarker stratification (CDH2^high/miR-210^high or EV-PD-L1 dynamics) must reproducibly segregate survival outcomes across at least two independent cohorts. Only upon fulfilling both tiers should RAX-targeted interventions progress to biomarker-enriched early-phase clinical trials. These criteria prevent premature escalation and anchor the ambitious scope of the RAX framework to measurable decision points.

## 28. Outstanding Questions and Cross-Disease Relevance

Several critical questions remain unresolved. Orthotopic tumors engineered to express HyPer (H_2_O_2_ sensor) could capture ROS pulses, while a Z-cad-style EMT/MET reporter and a CDH2 knock-in tag would read out TF activation and the CDH1-to-CDH2 switch in the same cell [106]. HyPer probes have been validated for dynamic H_2_O_2_ imaging in living cells and in vivo models [216,217,218,219], while single-cell transcriptomic analysis confirm that EMT trajectories generate hybrid EM states enriched for CSC traits and paracrine signaling [215]. Such integrated tri-reporter systems, coupled with intravital microscopy, would allow causal relationships between redox dynamics, transcriptional reprogramming, and adhesion remodeling to be established in vivo.

Another unresolved question is what encodes exosome cargo choice. Tumor-specific CRISPR knockouts or point mutants of YBX1, HNRNPA2B1, or ALIX (e.g., SUMO-site variants) could test their roles in exosomal miR-21, miR-200, miR-210 and PD-L1 loading, and the resulting effects on migration, T cell cytotoxicity, and TAM polarization. YBX1 has been identified as a key RNA-binding protein that selectively sorts miRNAs into exosomes, while HNRNPA2B1 directly regulates RNA cargo loading through post-translational modifications and has been validated by CRISPR knockout approaches in tumor models. In addition, ALIX functions within the ESCRT machinery to coordinate exosome biogenesis and cargo specificity [220,221]. Together, these mechanisms provide a strong genetic framework to test how exosome-mediated communication drives CSC plasticity and immune remodeling within the RAX hub.

A third question is whether hybrid E/M hubs are universal. Combined RNAscope and immunofluorescence for CDH2, AXL, FAK, PD-L1, and miR-210, integrated with spatial transcriptomics, could map hybrid E/M niches and EV-linked immune states across tumor stages. Recent spatial and imaging studies indicated that hybrid E/M phenotypes persist as niche-organized states enriched for CSC traits and immune-evasive programs; in colon cancer, phenotypic plasticity and niche-driven EMT dynamics are highlighted in comprehensive reviews of metastatic formation [218]. These findings underscore the feasibility of mapping hybrid niches across cancers to test the generalizability of RAX-driven plasticity.

They exhibit intrinsic plasticity, NCSC-like programs, and EV-mediated immune editing, together with tolerogenic macrophage states and checkpoint signaling, converging on exosomal PD-L1/miRNA immunomodulation [39,187]. Beyond these, PDAC demonstrates cadherin switching—loss of CDH1 with gain of CDH2—AXL/integrin/FAK signaling, reliance on GPX4/xCT redox buffering, and exosome-driven stromal and immune remodeling [222]. Similarly, colorectal and gastric cancers exploit exosomal miR-200 to reinforce epithelial plasticity, while miR-21 and miR-210 mediate hypoxic adaptation and immune suppression, correlating with metastasis and poor prognosis [223,224,225,226]. In TNBC, CDH2, AXL, and FAK signaling sustain invasive traits, while GPX4 and xCT protect CSC-like cells from ferroptosis; exosome cargo carrying PD-L1 and miRNAs further transfer drug resistance [216,227]. Importantly, systemic comorbidities such as diabetes converge on redox regulators including GPX4, linking metabolic stress to enhanced RAX reliance across multiple cancers.

## 29. Translational Outlook and Validation Roadmap

The RAX model yields biomarker–intervention loops that connect mechanistic discovery to translational application. Circulating exosomal CDH2, miR-200, and miR-210 emerge as minimally invasive biomarkers for hub activity. EMT-high/NCSC-high tumors such as melanoma, neuroblastoma, and subsets of lung cancers represent prime stratification candidates. Rational therapeutic strategies will require simultaneous disruption of adhesion (CDH2/AXL–FAK), redox buffering (GPX4/SLC7A11, FSP1, DHODH), and exosomal signaling to overcome redundancy and collapse CSC resilience.

To operationalize this framework, we propose a five-tier validation roadmap. First, causality can be interrogated using orthotopic tumors engineered with HyPer H_2_O_2_ sensors, EMT reporters, and CDH2 fluorescent knock-ins, enabling dynamic visualization of ROS-driven EMT/NCSC transitions. HyPer probes have been validated for dynamic H_2_O_2_ imaging in living cells and in vivo models [216,217,218,219], while single cell transcriptomic analysis confirm that EMT trajectories generate hybrid EM states enriched for CSC traits and paracrine signaling [215]. Intravital microscopy could integrate these reporters to resolve causality in vivo.

Second, pharmacologic buffering of ROS pulses using catalase mimetics or myeloperoxidase (MPO) inhibitors has been shown to modulate oxidative bursts in vivo [228]. Conversely, blockade of FAK/AXL signaling disrupts cadherin–integrin coupling and EMT-associated adhesion remodeling. Integrating these approaches, we propose that applying catalase mimetics versus adhesion pathway inhibitors in the same model would reveal whether pulse-to-adhesion coupling is redox-gated, directly testing the causal role of oxidative dynamics in EMT/NCSC stabilization [141,142,143,144,145].

Tumor-specific CRISPR knockouts or point mutants of YBX1, HNRNPA2B1, or ALIX (e.g., SUMO-site variants) could test their roles in exosomal miR-21, miR-200, miR-210 and PD-L1 loading, and the resulting effects on migration, T cell cytotoxicity, and TAM polarization [220]. YBX1 has been identified as a key RNA-binding protein that selectively sorts miRNAs into exosomes, while HNRNPA2B1 directly regulates RNA cargo loading through post-translational modifications and has been validated by CRISPR knockout approaches in tumor models. In addition, ALIX functions within the ESCRT machinery to coordinate exosome biogenesis and cargo specificity [221]. Together, these findings provide a mechanistic basis for testing how cargo selectors drive exosome-mediated plasticity and immune remodeling in the RAX hub.

Combined RNAscope and immunofluorescence for CDH2, AXL, FAK, PD-L1, and miR-210, integrated with spatial transcriptomics, could map hybrid E/M niches and EV-linked immune states across tumor stages [229]. Recent spatial transcriptomic and imaging studies confirm that hybrid E/M phenotypes are not transient but persist as niche-organized states enriched for CSC traits and immune-evasive programs. For example, Sahoo et al. demonstrated that hybrid E/M states sustain high PD-L1 levels and immune suppression, while Teeuwssen and colleagues highlighted the role of phenotypic plasticity and niche-driven EMT plasticity in metastatic progression in colon cancer models [230,231].

Finally, therapeutic stress tests should combine inhibition of CDH2/AXL–FAK axis (e.g., AXL ± FAK inhibitors) together with ferroptosis defenses (GPX4 or xCT blockade), and optionally suppress EV biogenesis (Rab27a, ESCRT). Readouts should include lipid-ROS accumulation/ferroptosis markers, CSC frequency, EV cargo flux (PD-L1, miR-21, miR-200, miR-210), and intratumoral T/NK cell function. This design is supported by evidence that FAK/AXL targeting disrupts mesenchymal programs and can improve response to anti-PD-1, while GPX4, xCT inhibition sensitizes mesenchymal/CSC-like cells to ferroptosis; EV output (Rab27a, ESCRT) sustains immune evasion via PD-L1 cargo, providing complementary leverage points [65,231,232].

Preclinical studies substantiate this concept: genetic or pharmacologic inhibition of the FAK axis reduces mesenchymal stability and significantly enhances anti-PD-1 efficacy with greater CD8^+^ T cell infiltration in vivo [233,234]. Similarly, blockade of TAM/AXL signaling suppresses EMT-associated invasion and confers immuno-oncologic benefit in animal models [233,235]. On the redox arm, disabling GPX4 or SLC7A11 elevates lipid peroxides and selectively triggers ferroptotic death in mesenchymal/CSC-like populations, effects that are reversed by ferrostatin-1 rescue. Finally, vesicle biogenesis via Rab27a, ESCRT machinery is essential for the export of exosomal PD-L1 and miRNA, sustaining T cell suppression; blocking these nodes diminishes immune evasion and sensitizes tumors to checkpoint blockade [220,221,236,237,238].

**Figure 3:**
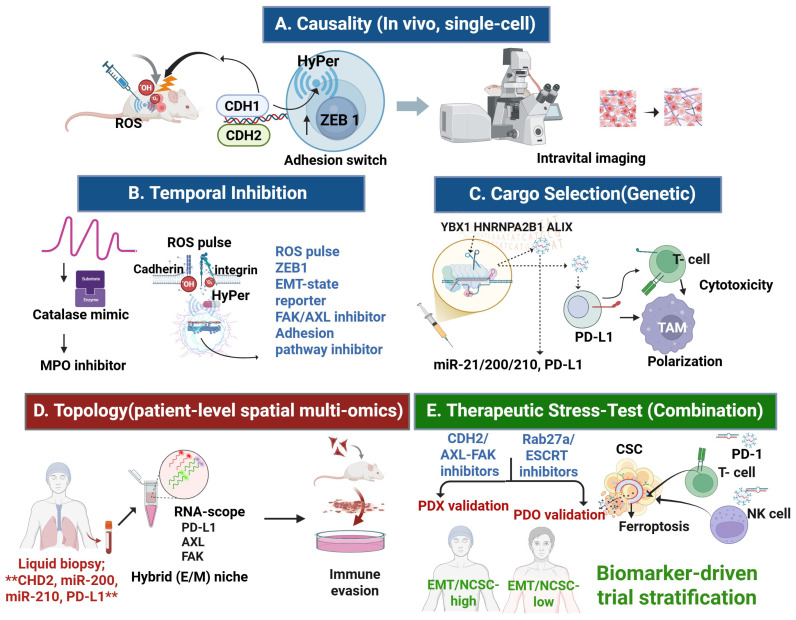
Experimental and translational validation roadmaps for the CDH2-centered redox–adhesion–exosome (RAX) hub. (**A**) Causality (in vivo, single-cell): Orthotopic tumors engineered with HyPer (H_2_O_2_ sensor), a ZEB1 EMT-state reporter, and a CDH2 knock-in tag enable real-time visualization of ROS pulses, transcription factor activation, and CDH1→CDH2 adhesion switching, linking oxidative bursts to CSC enrichment. (**B**) Temporal inhibition: ROS buffering with catalase mimetics or myeloperoxidase (MPO) inhibitors suppresses oxidative pulses, while FAK/AXL inhibition disrupts cadherin–integrin coupling, jointly testing whether ROS dynamics gate adhesion remodeling. (**C**) Cargo selection (genetic): CRISPR-mediated knockout of exosomal cargo selectors (YBX1, HNRNPA2B1, ALIX) alters loading of miRNAs (miR-21, miR-200, miR-210) and PD-L1, thereby reshaping tumor migration, T cell cytotoxicity, and macrophage polarization (TAM). (**D**) Topology (patient-level spatial multi-omics): Integration of RNAscope and multiplex immunofluorescence for CDH2, AXL, FAK, PD-L1, and miR-210 with spatial transcriptomics maps hybrid epithelial/mesenchymal (E/M) niches enriched for CSC traits and immune evasion signatures. (**E**) Therapeutic stress test (combination): In EMT-high tumors, concurrent inhibition of CDH2/AXL–FAK adhesion, GPX4 and xCT-mediated ferroptosis defenses, and Rab27a/ESCRT-dependent EV biogenesis reduces CSC frequency, enhances lipid–ROS accumulation, depletes exosomal PD-L1/miRNA cargo, and augments cytotoxic T lymphocyte (CTL) and natural killer (NK) cell infiltration. ** indicate key biomarkers included in the liquid-biopsy RAX panel. Arrows indicate direction of regulation: ↓ downregulation, ↑ upregulation. Abbreviations: AXL: AXL receptor tyrosine kinase; ALIX: ALG-2 interacting protein X; CDH2: cadherin 2; also known as N-cadherin; CSC: cancer stem cell; E/M: epithelial/mesenchymal; FAK: focal adhesion kinase; GPX4: glutathione peroxidase 4; HNRNPA2B1: heterogeneous nuclear ribonucleoprotein A2/B1; HyPer: hydrogen peroxide sensor probe; miR: microRNA; MPO: myeloperoxidase; NK cell: natural killer cell; PD-L1: programmed death-ligand 1; PDO: patient-derived organoid; PDX: patient-derived xenograft; ROS: reactive oxygen species; RNAscope: RNA in situ hybridization technology; SLC7A11: solute carrier family 7 member 11, also known as xCT, TAM: tumor-associated macrophage; ZEB1: zinc finger E-box binding homeobox 1; YBX1: Y-box binding protein 1. Created in BioRender. Kim, B. (2025) (License number: TC292KY13P).

This roadmap integrates causality testing, temporal inhibition, genetic dissection of cargo selection, spatial mapping of hybrid niches, and therapeutic stress-testing. Together, these strategies directly link mechanistic validation to biomarker development and clinical translation. In parallel, a composite liquid-biopsy panel—exosomal CDH2, miR-200, and miR-210—combined with tissue immunophenotyping (CDH2 ↑/CDH1 ↓; lineage markers as appropriate) enables longitudinal tracking and patient stratification (Figure 3A,B). Building on preclinical strategies (Table 2) and patient-cohort analyses (Table 3), we outline a staged validation roadmap (Table 4) that links mechanistic dissection to biomarker-guided clinical translation. This roadmap aligns with broader bench-to-bedside lessons from cancer nanomedicine, underscoring the need for stepwise validation [239]. Therapeutic targeting of lipid peroxidation dynamics—via ferroptosis inducers or antioxidant modulators—represents a direct translational extension of the RAX framework within the redox oncology paradigm.

Although our analysis highlights melanoma, neuroblastoma, and SCLC as prototypic EMT/NCSC-driven malignancies, similar associations are observed in colorectal and gastric cancers. For instance, miR-210 overexpression correlates with poor overall survival in CRC cohorts, while CDH2 upregulation predicts inferior prognosis in gastric cancer patients. These convergent signals across diverse epithelial tumors underscore the cross-disease generalizability of the RAX hub framework and support its broader translational potential. Patients stratified by combined CDH2^high/miR-210^high expression could be prioritized for RAX-targeted intervention trials, analogous to PD-L1 or tumor mutational burden (TMB)-based enrichment in immunotherapy trials [176,177,241,242,243,244,245]. Across melanoma patient cohorts, exosomal PD-L1 levels consistently predicted inferior outcomes under ICIs, where higher EV PD-L1 abundance was linked to shorter overall survival and reduced therapeutic efficacy. Serial monitoring of EV PD-L1 further enabled discrimination between durable responders and early progressors [246,247,248], supporting its role as a putative predictive biomarker for biomarker-guided RAX-targeted interventions.

Tissue/EV miR-200 low/ZEB1 high EMT axis correlates with adverse survival across multiple cancers, as supported by meta-analysis and NSCLC cohort data [249]. Exosomal miR-210 reflects hypoxia- and redox-driven adaptation, and its elevation has been associated with tumor progression and poorer clinical outcomes in NSCLC and PDAC cohorts [250]. On the adhesion arm, CDH2 expression consistently associates with aggressive behavior and reduced survival across tumor types; moreover, genetic reduction in CDH2 prolongs survival in a KPC pancreatic cancer model, underscoring its prognostic and therapeutic relevance [251,252].

Clinically, activation of the AXL/FAK signaling pathway has been repeatedly linked to poor prognosis and therapeutic resistance and is currently the focus of multiple translational studies and clinical trials [253,254]. Together, these literature-derived clinical associations support biomarker-guided enrichment strategies—particularly panels combining exosomal CDH2, miR-200, miR-210, and EV-PD-L1—for precision RAX-targeted interventions [255]. Pan-cancer meta-analyses demonstrate that low miR-200 and high ZEB1 expression correlate with EMT activation, enhanced invasion, and significantly worse overall and disease-free survival across multiple tumor types, supported by TCGA-based pan-cancer datasets [256]. Emerging clinical evidence further highlights the translational relevance of EV-associated microRNAs in RAX-dependent cancers. In particular, EV miR-210 has been associated with hypoxia-conditioned redox adaptation, metastatic progression, and unfavorable outcomes in observational analyses of PDAC and non-small-cell lung cancer (NSCLC) cohorts. In PDAC, serum-derived EV miR-210 levels are significantly elevated compared with controls and associate with early detection as well as adverse prognosis [257]. Complementary mechanistic and clinical analyses confirm that miR-210 functions as a central hypoxia/redox-responsive oncomiR that promotes invasion, angiogenesis, and therapy resistance [258]. Serial changes in EV PD-L1 (±miR-210) were associated with response status in exploratory NSCLC cohorts, supporting their potential predictive utility; however, prospective validation with standardized assays and predefined thresholds is required [249,250]. Standardized assays and pre-specified thresholds will be essential to determine whether these EV markers function as predictive rather than purely prognostic indicators. Collectively, these findings support the integration of EV miR-210 quantification into liquid-biopsy panels, in combination with exosomal CDH2 and miR-200 family members, to refine patient stratification for RAX-targeted therapeutic strategies.

Across cancers, N-cadherin (CDH2) upregulation is consistently linked to aggressive disease biology and poor survival outcomes. A large meta-analysis confirmed that elevated CDH2 predicts inferior overall survival and higher risk of metastasis in epithelial-derived tumors [251,259]. Importantly, genetic reduction in Cdh2 in a KPC pancreatic cancer model prolonged survival by ~25%, providing in vivo evidence that targeting CDH2 can improve prognosis [252]. Clinically, the AXL–FAK axis represents a conserved driver of resistance and poor prognosis across multiple tumor types [260]. AXL overexpression promotes epithelial–mesenchymal transition (EMT), enhances cell migration/invasion, and sustains therapy resistance, while its crosstalk with FAK amplifies integrin signaling and adhesion remodeling [260,261]. Evidence from NSCLC, TNBC, RCC, and PDAC demonstrates that AXL activation correlates with adverse survival and immune evasion, positioning it as both a biomarker of poor prognosis and a therapeutic target [260]. Preclinical studies show that combined AXL and FAK inhibition disrupts mesenchymal stability, restores drug sensitivity, and synergizes with anti-PD-1 therapy, leading to enhanced CD8^+^ T cell infiltration [261]. Ongoing clinical trials of AXL inhibitors (e.g., bemcentinib, cabozantinib) underscore the translational relevance of this pathway [262]. Together, integrating exosomal CDH2, miR-200/miR-210, and EV-PD-L1 with AXL–FAK activation provides a composite stratification framework for identifying RAX hub-dependent tumors most likely to benefit from multi-node therapeutic disruption (Figure 3; Table 5). This aligns with emerging perspectives that move beyond the genetic paradigm of cancer, emphasizing cell-state plasticity and tissue-level field effects as central drivers of malignancy [240]. As summarized in Table 5, recent landmark studies emphasize the clinical feasibility of biomarker-guided strategies, the conceptual shift beyond the genetic paradigm [240], and the necessity of dynamic, patient-specific modeling approaches [182,190]. Collectively, these findings substantiate the RAX hub as a field-shaping and translationally actionable framework.

## 30. Conclusions

Cancer progression is driven less by static hierarchies than by dynamic plasticity. Here, we integrate developmental and oncogenic paradigms into a single framework: an EMT/MET–neural crest convergence anchored by a CDH2-centered redox–adhesion–exosome (RAX) hub. In this model, transient ROS pulses license EMT/NCSC transcriptional switches; cadherin switching and integrin–FAK/Src signaling establish migratory adhesion states; exosomes propagate mesenchymal, redox, and immunoregulatory programs; and redox-buffering circuits—NRF2–SLC7A11–GPX4 and FSP1–CoQ10/DHODH–CoQH2 axes—maintain oxidative balance. The outcome is stabilization of cancer stemness, metastatic competence, and immune evasion as a self-reinforcing circuit.

This perspective yields three translational opportunities. First, it nominates liquid-biopsy biomarkers—exosomal CDH2, miR-200, and miR-210—as minimally invasive, real-time reporters of hub activity. Second, it defines a biomarker-driven stratification schema: EMT-high/NCSC-high tumors, including melanoma, neuroblastoma, small-cell lung cancer, and subsets of pancreatic and triple-negative breast cancers, are predicted to be most RAX-dependent and thus therapeutically targetable. Third, it proposes a therapeutic blueprint: rather than single-node inhibition, rational multi-node combinations (e.g., CDH2, AXL–FAK inhibitors plus GPX4, xCT ferroptosis blockade and exosome suppression) may collapse both intrinsic buffering and extrinsic niche programming.

Operationalizing this strategy requires translational pipelines: state-aware preclinical models using EMT sensors, organoids, and patient-derived xenografts, prospective validation of the exosomal CDH2/miR-200/miR-210 biomarker panel in liquid-biopsy cohorts, and early-phase clinical trials enriched for EMT/NCSC-high patients, with biomarker-guided escalation across adhesion, redox, and exosome axes. Key open questions include the quantitative rules of ROS pulsing, the logic of exosomal cargo selection, and resistance trajectories under multi-node therapeutic pressure.

Collectively, the RAX hub may redefine metastasis and therapy resistance as emergent network properties, proposing a biomarker-guided roadmap for precision oncology. By measuring and dismantling this network rather than chasing single effectors, we anticipate a path toward translational frameworks for multi-target redox and exosome modulation in metastatic cancers, in which metastatic and immune-evasive phenotypes become not only predictable but also clinically actionable—and ultimately preventable.

## Figures and Tables

**Table 1:** Translational and mechanistic features distinguishing the CDH2-centered RAX hub from conventional EMT/redox/exosome models.

Theme	Scope	Gap/Limitation	Contribution (RAX Model)	Evidence	Translation	Refs.
EMT plasticity and hybrid E/M state	EMT spectrum; invasion/metastasis; CSC emergence; EMT-TFs (TWIST, ZEB, SNAIL); epigenetic regulators	No ROS licensing; no CDH2 hub; weak EMT–EV/immune coupling	RAX closed-loop; CDH2-centered hub; EMT/NCSC transitions linked to EV–immune axis	Preclinical/conceptual (single-cell; lineage tracing; HyPer imaging potential)	EMT/NCSC-high tumors (TNBC, PDAC, neuroblastoma); EMT + CDH2 biomarker panels	[57,58,59]
CDH2/AXL–FAK adhesion axis	FAK as integrator of integrin/growth factor signals; EMT induction; angiogenesis; immunosuppressive TME; AXL as TAM RTK driving EMT, metastasis, drug resistance	Linear pathway perspective; no systems-level integration; weak linkage to ROS dynamics; limited exosomal cargo context	CDH2 as adhesion hub; feedback to redox buffering; EV cargo selection; FAK/AXL within closed RAX circuit; governs invasion, immune evasion, metabolic remodeling	Preclinical/clinical; multiple drug reports; AXL/FAK inhibitors in lung, breast, pancreatic cancers; early trials	Adhesion axis inhibition as one pillar of therapeutic triad (with ferroptosis induction + EV blockade); RAX-stratified tumors (CDH2–FAK/AXL-high)	[60,61,62,63]
Redox buffering (NRF2–SLC7A11/GPX4; FSP1–DHODH)	Redox/ferroptosis defense reviews; canonical system Xc^−^/GSH/GPX4; non-canonical FSP1/DHODH, TRX system; NRF2/xCT/GPX4 as core survival axes	Ferroptosis defenses treated in isolation; no linkage to EMT licensing, adhesion remodeling, or exosomal immune escape; weak systems perspective	Redox buffering as “R” arm; licenses CDH2–FAK adhesion remodeling; enables exosome-mediated immune reprogramming; reframes ferroptosis-resistance as driver of CSC plasticity and immune evasion	Omics, CRISPR, in vivo studies; NRF2–SLC7A11–GPX4 axis → ferroptosis- resistance + immune exclusion; FSP1/DHODH → mitochondria–CoQ–lipid buffering; TRX system → redox–ferroptosis crosstalk; ferroptosis–immune interactions in cervical and other cancers	Druggable ferroptosis nodes: GPX4, SLC7A11, FSP1, DHODH, TRX2; Biomarkers: GPX4, SLC7A11, NRF2 activity for patient stratification and treatment response	[64,65,66,67,68]
Exosome cargo selection and PD-L1/miRNA circuits	EV/immune checkpoint reviews; cargo selection via YBX1, hnRNPA2B1, ALIX; ESCRT machinery; exosomal PD-L1; immune-regulatory miRNAs (miR-21, miR-155, miR-200 family, miR-210)	Siloed analyses of EV biogenesis and immune suppression; weak integration with adhesion (CDH2/EMT spectrum) or ROS pulse licensing; limited link to systemic immune escape	Cargo selection bound to EMT/CDH2 states and ROS-driven licensing; exosomal PD-L1 and miR-200/210 as dynamic readouts of RAX loop (adhesion–ferroptosis–immune coupling)	Exosomal PD-L1 → CTL suppression and immunotherapy resistance; exosomal miRNAs (miR-155, miR-21, miR-200, miR-210) → invasion, angiogenesis, immune escape; liquid-biopsy detection of exosomal miRNAs (miR-29, miR-34, miR-203, miR-378) and PD-L1 feasible for treatment monitoring	EV biogenesis blockade = third therapeutic pillar (with ferroptosis induction + adhesion inhibition); liquid-biopsy panels (exosomal PD-L1 + ROS-responsive miRNAs) for patient stratification and monitoring	[69,70,71,72,73,74,75]
Immune evasion ↔ ferroptosis coupling	Immune–redox crosstalk reviews; ROS/redox regulators (NRF2, GLRX, TRX); ferroptosis defenses (NRF2–SLC7A11–GPX4, FSP1/DHODH); PD-L1/MHC regulation; cytokine signaling	Descriptive, pathway-specific coupling; no closed-loop model; ferroptosis defenses not mechanistically linked to checkpoint evasion, EMT plasticity, or EV suppression	NRF2-driven PD-L1 induction; MHC-I loss; EV cytokine/miRNA circuits mapped to ferroptosis defense; redox buffering enables immune evasion + CSC survival in closed RAX loop	ROS licensing of EMT/adhesion/checkpoints; GLRX and NRF2 correlate with PD-L1 and macrophage suppression in glioma; CSC plasticity + checkpoint resistance under redox stress/hypoxia; NSCLC and melanoma → PD-L1 upregulation with ferroptosis-resistance	Combination: anti-PD-1/PD-L1 + adhesion inhibition + ferroptosis induction + EV blockade; RAX-high tumors (EMT + CDH2 + NRF2 signatures; exosomal PD-L1/miRNA readouts)	[23,24,28,76,77,78,79,80]
Spatial and liquid-biopsy readouts	Translational biomarker reviews; spatial transcriptomics; single-cell omics; cfDNA; CTCs; exosomal profiling; candidate biomarkers for detection/ monitoring	Fragmented focus; no integrative multi-axis signature; absence of RAX-type closed- circuit frameworks	Minimal RAX panel: CDH2, EMT markers, GPX4/xCT, exosomal PD-L1/miR-200/210; integrated decision flow linking adhesion, redox, and EV axes for patient stratification	Clinical/translational; supported by single-cell/spatial omics; cfDNA; EV-based liquid biopsy; feasibility shown for early detection and dynamic monitoring	RAX-guided clinical trial stratification: EMT + CDH2 + redox + EV biomarker panels; real-time patient monitoring via cfDNA + exosomal PD-L1/miRNAs; decision tree framework	[81,82,83,84,85,86]
Therapeutic triad strategy	List combinations within single domains	Seldom justify triads via a systems closed-loop model	Justifies adhesion axis inhibition + ferroptosis induction + EV blockade as loop- disruptive triad	Preclinical/ conceptual	Designs synthetic lethality-style trials and pharmacodynamic readouts per pillar	
Validation roadmap (4-step)	Describe tools separately	No end-to-end plan linking imaging → cargo selectors → spatial multi-omics → clinical panel	Provides staged roadmap: HyPer–EMT–CDH2 imaging → cargo- selector (YBX1/hnRNPA2B1/ALIX) CRISPR → spatial multi-omics → liquid-biopsy	Preclinical→ translational (progressive)	De-risks clinical translation with measurable milestones and Go/No-Go criteria	[87,88,89,90,91,92,93,94]

Scope summarizes the major concepts addressed in representative prior reviews. Gap/Limitation highlights missing systems-level connections (e.g., absence of ROS licensing mechanisms or EV–immune coupling). Contribution (RAX model) describes how the proposed CDH2-centered redox–adhesion–exosome (RAX) hub integrates adhesion, redox, and extracellular-vesicle (EV) axes into a closed regulatory loop. Evidence is categorized as preclinical/conceptual (e.g., single-cell analyses or intravital imaging feasibility) versus clinical/translational (e.g., patient cohorts or early-phase clinical trials). Translation lists actionable readouts—such as biomarkers or imaging parameters—foreseeable for patient stratification or therapeutic monitoring. Abbreviations: AXL: AXL receptor tyrosine kinase; CDH2: Cadherin-2 (N-cadherin); cfDNA: cell-free DNA; CSC: cancer stem cell; CTC: circulating tumor cell; CTL: cytotoxic T lymphocyte; DHODH: dihydroorotate dehydrogenase; EMT: epithelial–mesenchymal transition; EV: extracellular vesicle; FAK: focal adhesion kinase; FSP1: ferroptosis suppressor protein 1; GPX4: glutathione peroxidase 4; NRF2: nuclear factor erythroid 2-related factor 2; PD-L1: programmed death-ligand 1; SLC7A11; xCT: solute carrier family 7 member 11 cystine/glutamate antiporter; TME: tumor microenvironment; TRX: thioredoxin. Direction indicates the predominant functional effect summarized from the cited literature (e.g., promotes EMT, stabilizes CSC traits, enhances immune evasion). See text for context and exceptions.

**Table 2:** Multi-node therapeutic combinations across adhesion, redox, and exosome axes: pharmacodynamic endpoints and design considerations.

Axis Combination	Example Agents (Class)	Primary PD Readouts (Pre-Specified)	Trial Design Considerations	Key Findings/Rationale
Adhesion + Redox	AXL inhibitor (TP-0903), FAK inhibitor (Y15), ± ErbB blockade (Afatinib)	p-FAK(Y397) ↓, E→N-cadherin switch, invasion ↓	Biomarker-enriched cohorts (CDH2^high/AXL^high, GPX4/SLC7A11 signatures); dose-finding sequencing.	Low-dose AXL/FAK/ErbB triple blockade synergistically reduced survival, migration, and EMT markers in HNC models [165].
Redox (ferroptosis)	GPX4 inhibitor (RSL3, ML162), SLC7A11 blockers (erastin)	Lipid ROS ↑ (BODIPY-C11), GPX4 activity ↓	Patient stratification for ferroptosis sensitivity; ferroptosis rescue assays	GPX4 inhibition or SLC7A11 blockade sensitizes tumors to ferroptosis; SLC7A11 shown to upregulate PD-L1, linking ferroptosis defense to immune evasion [166,167].
Redox + EV	GPX4/SLC7A11 inhibition + EV biogenesis/release inhibition or EV-PD-L1 neutralization	Lipid ROS ↑, EV-PD-L1 ↓, miR-210 ↓/miR-200 ↑	On-treatment liquid-biopsy monitoring; assay standardization and predefined thresholds.	EVs modulate ferroptosis-linked death pathways; longitudinal EV-PD-L1 dynamics in NSCLC stratified ICI benefit and survival [168].
EV cargo miRNAs	Exosomal miR-210 modulation	Hypoxia-driven adaptation, STAT3/PI3K/AKT pathways	Tumor-type-specific miRNA panels; integration with hypoxia and immune signatures.	Lung CSC-derived exosomal miR-210-3p promoted migration/invasion/EMT and metastasis via FGFRL1 targeting [169,170].
Adhesion + EV	Anti-CDH2 antibody, AXL interference + EV suppression	EMT/NCSC score ↓, EV flux ↓, CD8^+^ T cell cytotoxicity ↑	Combine AXL/FAK interference with EV suppression; consider ICI add-on in expansion cohorts contingent on PD readouts.	Conceptual support for combining adhesion blockade with EV pathway inhibition and immune modulation [165].

Arrows indicate direction of regulation: ↓ downregulation, ↑ upregulation. Biomarker source denotes tissue-based versus liquid-biopsy extracellular vesicle (EV) measures; EV isolation and quantification methods should be reported (e.g., size-exclusion chromatography or ultracentrifugation for isolation; nanoparticle tracking analysis (NTA) for particle counts; ELISA or parallel reaction monitoring (PRM) for protein assays; RT-qPCR or ddPCR for miRNA quantification). Primary pharmacodynamic (PD) readouts are prespecified assay endpoints per axis: adhesion (p-FAK^Y397, EMT score, E→N cadherin switch); redox (BODIPY-C11 lipid-ROS, GPX4 activity); and EV signaling (EV-PD-L1, EV miR-210/miR-200). Outcome/Direction: “Predictive” denotes association with treatment benefit (e.g., immune-checkpoint inhibitor (ICI) response, progression-free survival (PFS), or overall survival (OS) under therapy); “Prognostic” indicates outcome irrespective of therapy; “Adverse” refers to unfavorable directionality (e.g., miR-200 ↓/ZEB1 ↑, CDH2 ↑). N indicates the sample size in the cited cohort or meta-analysis when available; if multiple cohorts exist, the largest or most representative is reported. Statistics (hazard ratio [HR], odds ratio [OR], area under curve [AUC], confidence interval [CI]) and cut-offs are study-specific and not harmonized across cohorts; prospective trials should prespecify thresholds and assay quality control (QC). Safety sequencing and de-escalation rules are recommended for multi-node regimens (e.g., initiate with adhesion axis inhibition, add ferroptosis induction upon PD failure, and include EV blockade contingent on EV-PD-L1 dynamics). ICI add-on in expansion cohorts should be considered when EV-PD-L1 levels decrease and intratumoral CD8^+ PD readouts improve. Abbreviations: AXL: AXL receptor tyrosine kinase; CDH2: Cadherin-2 (N-cadherin); EV: extracellular vesicle; FAK: focal adhesion kinase; GPX4: glutathione peroxidase 4; ICI: immune checkpoint inhibitor; NCSC: neural crest stem-like cell; PD: pharmacodynamic; PD-L1: programmed death-ligand 1; ROS: reactive oxygen species; SLC7A11 (xCT): solute carrier family 7 member 11, cystine/glutamate transporter.

**Table 3:** Clinical cohort evidence linking CDH2 and hypoxia-regulated miR-210 expression to survival outcomes and therapeutic resistance, highlighting their integration into the RAX hub framework.

Axis/Biomarker	Clinical Cohort Evidence	Key Findings/Rationale	Refs.
miR-210 (hypoxia-linked)	Clinical cohorts: TCGA colorectal cancer; breast cancer; lung cancer; PDAC datasets	High miR-210 expression correlates with poor overall survival; hypoxia-induced adaptation; associated with EMT and therapeutic resistance	[176,177]
CDH2 (N-cadherin)	Clinical datasets: TCGA pan-cancer; breast and lung cohorts	Elevated CDH2 expression predicts inferior OS and PFS; correlates with EMT-high tumors; supports RAX hub role in mesenchymal stabilization	[178,179]
Combined CDH2^high + miR-210^high	Clinical cohorts: TCGA colon, breast, and lung cancer datasets	Double-high group exhibits the worst prognosis compared to other combinations; mechanistically links adhesion remodeling and hypoxia-driven miRNA signaling	[180]

Clinical cohort evidence is derived from TCGA and published clinical datasets, with re-analyses focusing on epithelial–mesenchymal transition (EMT) and hypoxia-related transcriptional signatures. Combined CDH2^high + miR-210^high signature is based on integrated TCGA and Gene Expression Omnibus (GEO) meta-re-analyses, demonstrating an additive prognostic impact on survival outcomes across multiple cancer types. Abbreviations: CDH2: Cadherin-2 (N-cadherin); EMT: epithelial–mesenchymal transition; GEO: Gene Expression Omnibus; miRNA: MicroRNA; OS: overall survival; PDAC: pancreatic ductal adenocarcinoma; PFS: progression-free survival; RAX hub: redox–adhesion–exosome hub; TCGA: The Cancer Genome Atlas.

**Table 4:** Redefining cancer plasticity: A translational and paradigm-shifting framework.

Key Findings	Relevance to RAX Hub Framework	Ref.
Highlights the central role of biomarkers in diagnosis, therapeutic decision-making, and disease monitoring in oncology	Supports the clinical feasibility of exosomal CDH2/miRNA panels as diagnostic and predictive biomarkers within the RAX hub.	[181]
Developed a biomarker discovery pipeline integrating TCGA expression and DepMap survival data, identifying robust progression gene signatures validated across multiple cohorts.	Demonstrates the power of integrating omics and survival datasets, reinforcing the rationale for exosomal CDH2/miR-210 as a prognostic progression signature.	[186]
Reviews translational pathways of nanomedicine, outlining challenges and success stories in clinical implementation.	Provides translational precedent for the RAX hub therapeutic triad (adhesion inhibition, ferroptosis induction, exosome blockade) as a bench-to-bedside strategy.	[239]
Argues for a shift beyond the genetic paradigm, emphasizing cell-state plasticity and tissue-level field effects in cancer progression.	Conceptually aligns with the RAX hub as a closed-loop model integrating redox licensing, adhesion switching, and exosomal field reprogramming.	[240]
Proposes dynamic, feedback-driven therapeutic approaches to address cancer evolution and resistance.	Resonates with the RAX hub therapeutic roadmap, emphasizing multi-node, adaptive interventions against EMT plasticity and ferroptosis defense.	[182]
Reviews mechanistic + AI-integrated models enabling patient-specific treatment prediction and optimization.	Reinforces the future applicability of RAX hub modeling to design biomarker-enriched, personalized therapeutic strategies.	[190]

Biomarker findings are derived from integrated analyses of TCGA and DepMap datasets, complemented by translational nanomedicine reviews and paradigm-integration frameworks in oncology. Conceptual alignment with the RAX hub emphasizes cell-state plasticity, adhesion switching, ferroptosis defense, and exosomal remodeling as interconnected mechanisms underlying therapeutic resistance and adaptive evolution. Abbreviations: AI, artificial intelligence; CDH2, Cadherin-2 (N-cadherin); EMT, epithelial–mesenchymal transition; miRNA, MicroRNA; RAX hub, redox–adhesion–exosome hub; TCGA, The Cancer Genome Atlas; DepMap, Dependency Map project.

**Table 5:** Clinical associations of RAX-hub biomarkers across cancers: evidence and paradigm-shifting implications.

Tumor Type	Biomarker (Tissue/EV)	N (Repr.)	Endpoint	Direction	Refs.
Melanoma	EV PD-L1	Multiple independent cohorts	ICI response/OS	Predictive for EV PD-L1 ↑ → ICI benefit ↓, OS shorter; longitudinal EV PD-L1 dynamics stratify durable responders vs. non-responders	[246,247,248,263,264]
NSCLC	Serum exosomal PD-L1	120 (meta-analysis)	High baseline exoPD-L1 → poor PFS; post-treatment exoPD-L1 downregulation → superior OS/PFS	Predictive for anti-PD-1 response/survival	[265]
NSCLC (validation)	EV-miR-625-5p (linked with PD-L1 status)	88	EV-miR-625-5p levels stratify patients with PD-L1 ≥ 50%, predictive of ICI response and survival	Predictive for durable benefit on ICIs	[249,253]
Pan-cancer	miR-200 family (↓)/ZEB1 (↑)	TCGA-based meta-analysis	OS/DFS	adverse	[255,266]
Lung/PDAC	EV miR-210	Clinical + Review evidence	Metastasis/OS	adverse	[249,250,257,258,267]
Pan-cancer	CDH2/N-cadherin (↑)	Meta/Review (multi-cohort, TCGA-based)	OS/metastasis	adverse	[268,269,270,271]
PDAC	CDH2/N-cadherin (↓)	Preclinical (KPC model)	OS	Prolonged survival	[252,272]
Multi-tumor (review)	AXL (±FAK)	Review/Trials	Therapy resistance, EMT induction, poor prognosis	adverse	[260,261,262]

Arrows indicate direction of regulation: ↓ downregulation, ↑ upregulation. Clinical cohort evidence is derived from independent patient datasets, TCGA-based meta-analyses, and exosome-focused biomarker studies, with emphasis on ICI response and survival outcomes. Exosomal PD-L1, miR-210, and CDH2 represent core RAX hub biomarkers linked to immune evasion, metastasis, and mesenchymal stabilization. Preclinical validation (KPC pancreatic cancer model) supports the functional relevance of CDH2 modulation in survival. Abbreviations: AXL: AXL receptor tyrosine kinase; CDH2: Cadherin-2 (N-cadherin); DFS: disease-free survival; EMT: epithelial–mesenchymal transition; EV: extracellular vesicle; FAK: focal adhesion kinase; ICI: immune checkpoint inhibitor; miR: microRNA; OS: overall survival; PD-L1: programmed death-ligand 1; PDAC: pancreatic ductal adenocarcinoma; PFS: progression-free survival; RAX: redox–adhesion–exosome; TCGA: The Cancer Genome Atlas; ‘adverse’ denotes higher risk or poorer outcome. Biomarker source: EV = extracellular vesicle; serum/plasma as specified. Representative references shown; see text for additional cohorts.

## Data Availability

No new data were created or analyzed in this study.

## Abbreviations

protein kinase B	(AKT)
ALG-2 interacting protein X	(ALIX)
AXL receptor tyrosine kinase	(AXL)
cancer stem cell	(CSC)
cell-free DNA	(cfDNA)
circulating tumor cell	(CTC)
clustered regularly interspaced short palindromic repeats	(CRISPR)
cadherin 2, also known as N-cadherin	(CDH2)
epithelial–mesenchymal transition	(EMT)
epithelial/mesenchymal	(E/M)
extracellular vesicle	(EV)
extracellular matrix	(ECM)
focal adhesion kinase	(FAK)
ferroptosis suppressor protein 1	(FSP1)
forkhead box D3	(FoxD3)
glutamate–cysteine ligase catalytic subunit	(GCLC)
glutathione	(GSH)
glutathione peroxidase 4	(GPX4)
heterogeneous nuclear ribonucleoprotein A2/B1	(hnRNPA2B1)
heme oxygenase-1	(HO-1)
HyPer hydrogen peroxide sensor probe	(HyPer)
kelch-like ECH-associated protein 1	(KEAP1)
leucine-rich repeat-containing G-protein coupled receptor 5 positive	(LGR5^+^)
mitogen-activated protein kinase	(MAPK)
myeloperoxidase	(MPO)
Msh homeobox 1	(MSX1)
NAD(P)H quinone dehydrogenase 1	(NQO1)
neural crest stem cell	(NCSC)
nuclear factor erythroid 2–related factor 2	(NRF2)
nuclear factor kappa-light-chain-enhancer of activated B cells	(NF-κB)
pancreatic ductal adenocarcinoma	(PDAC)
platelet-derived growth factor receptor beta	(PDGFRB)
programmed death-ligand 1	(PD-L1)
proto-oncogene tyrosine-protein kinase Src	(Src)
reactive oxygen species	(ROS)
RNA in situ hybridization technology	(RNAscope)
SRY-box transcription factor 10	(SOX10)
solute carrier family 7 member 11, also known as xCT	(SLC7A11, xCT)
small-cell lung cancer	(SCLC)
snail family transcriptional repressor 1	(SNAI1, Snail protein)
twist family bHLH transcription factor 1	(TWIST1, Twist protein)
transforming growth factor-β	(TGF-β)
Thioredoxin	(TRX)
triple-negative breast cancer	(TNBC)
Y-box binding protein 1	(YBX1)
zinc finger E-box binding homeobox 1	(ZEB1)
